# Association between BMI and health-related physical fitness: A cross-sectional study in Chinese high school students

**DOI:** 10.3389/fpubh.2022.1047501

**Published:** 2022-12-08

**Authors:** Guoyang Qin, Yong Qin, Bin Liu

**Affiliations:** ^1^College of Physical Education, Shandong Normal University, Jinan, Shandong, China; ^2^Physical Education Teaching and Research Office, Lixia District Bureau of Education and Sports of Jinan City, Jinan, Shandong, China; ^3^Physical Education Teaching and Research Group, Jinan Licheng No. 2 High School, Jinan, Shandong, China

**Keywords:** high school students, body mass index, physical fitness, cross-sectional study, China

## Abstract

**Background:**

Existing studies reporting on the levels of physical fitness among high school students use relatively few fitness tests for indicators of physical fitness, thus, incomprehensively evaluating the levels of physical fitness. Therefore, this study investigated the relationship between body mass index (BMI) and physical fitness index (PFI) by investigating five physical fitness indicators and calculating PHI.

**Method:**

Anthropometric measurements and indicators from five measures of physical fitness (50-m sprint, sit and reach, standing long jump, 800/1,000-m run, pull-up/bent-leg sit-up) were assessed. BMI was calculated to classify individuals into underweight, normal weight, overweight, and obese categories. *Z*-scores based on sex-specific mean and standard deviation were calculated, and the sum of *Z*-scores from the six fitness tests indicated the PFI. The findings were fitted to a linear regression model to elucidate the potential relationship between BMI and PFI.

**Results:**

In total, 176,655 high school students (male: 88,243, female: 88,412, age: 17.1 ± 1.05 years, height: 168.87 ± 11.1 cm, weight: 62.54 ± 15.15 kg) in Jinan, China, completed the physical fitness tests between 2020 and 2021. The one-way ANOVA models showed that PFI in the normal category was significantly higher as compared to all the other BMI categories within both male and female groups (*p* < 0.001), and PFI in the obese category was significantly lower as compared to all the other BMI categories for both male and female groups (*p* < 0.001). The association between PFI and BMI showed an inverted U-shape relationship.

**Conclusions:**

This study demonstrated that BMI affects the PFI in both males and females. As compared to the obese and overweight categories based on BMI, significantly higher scores of PFI were observed for males and females.

## Introduction

The prevalence of obesity among adolescents has been rising globally due to the accelerated rate of unhealthy eating, and reduced or lack of adequate physical activity, and has become a public health problem (1). The high school comprises the transitional period from adolescence to adulthood and is crucial for developing healthy lifestyles and forming healthy behaviors (2, 3). In recent years, there has been a significant decline in physical activity among high school students (1, 4, 5). A study have shown that reduced physical activity can lead to weight gain and increased prevalence in adolescents (6). Being overweight is becoming increasingly common in high school students, while the physical fitness of adolescents is on a decline. Moreover, the desire to be thin is common among young people in Asia (7, 8). These regions and countries face the dual burden of both underweight and overweight adolescents (9, 10). Therefore, weight monitoring to maintain good health is crucial for high school students.

Body mass index (BMI) is universally considered a marker of health and is widely used to measure malnutrition, overweight, and obesity (11–13). Studies have shown that an increase in BMI increases the risk of cardiovascular disease (hypertension, myocardial infarction, lung disease, sleep apnea syndrome) (14). Also, study have shown that BMI can effectively reflect the physical fitness of ordinary college students (15). Moreover, physical fitness correlates positively with physical activity (16). Some studies have discussed the association between BMI and several components of physical fitness in children (17) and adolescents (18). Also study suggested that there was a significant difference between the (BMI) of normal females compared to the scoliotic female high school student (19). Therefore, monitoring the BMI of high school students is of great significance to understanding their physical development (20). Studies have shown that BMI may be affected by various factors (i.e., ethnic groups) (20), so it is unclear whether using the BMI index reflects their physical fitness and health.

This study aimed to analyze the levels of different physical fitness components among high school students and evaluate the association between BMI and health-related physical fitness. We hypothesized that BMI could effectively reflect the physical fitness levels of Chinese high school students.

## Materials and methods

### Subjects

The data were collected from a national survey on physical fitness conducted among high schools between 2020 and 2021 in Jinan of Shandong province, China. Students aged 15–18 years completed the physical fitness tests (*n* = 176,655, male: 88,243, female: 88,412, age: 17.1 ± 1.05years, height: 168.87 ± 11.1 cm, weight: 62.54 ± 15.15 kg; see Supplementary Figure S1 for the recruitment process). Due to the pandemic, high school students were in relatively closed state, in which students were just in school and at home regularly. For all participants, both participants and their parents (or guardians) gave their informed consent. The study protocol complied with the Declaration of Helsinki and was approved by the Ethics Committee of Shandong Normal University (2021036).

### Procedures

According to the technical specifications including the “National Student Physical Health Standard,” we first conducted anthropometric measurements, followed by tests for various physical fitness indexes (PFIs), and finally the cardiorespiratory endurance test. In the standing long jump test, the “best of three jumps” was considered the result; “the best of two” results were considered for the 50-m running test, while other tests were performed once. We adopted intelligent physical health monitoring equipment, through non-contact measurement using the infrared multi-point sensor array, which automatically recorded the students' scores and uploaded these values to the system for storage.

### BMI calculation

The BMI was calculated using the following formula: BMI = weight (kg)/height (m)^2^. Students were divided into four categories based on their BMI values according to the criteria recommended by the World Health Organization (WHO) as follows: < 18.5 kg/m^2^, 18.5–23.9 kg/m^2^, 24–27.9 kg/m^2^, and ≥28 kg/m^2^, representing underweight, average weight, overweight, and obese individuals, respectively (21).

### Physical fitness test

The tests for physical fitness included 50 m sprints, sit and reach, standing long jump, 800/1,000 m runs, pull-ups, and bent-leg sit-ups.

#### 50 m sprint

To evaluate students' speed and explosive strength a 50 m sprint was conducted. Students were tested in groups of four. When the investigator indicated, “go,” the subjects began the 50 m sprint. They finished the run as fast as they could. The time in minutes and seconds was recorded (15).

#### Sit and reach

To assess lower hamstring flexibility, a sit and reach test was conducted. Each subject was barefoot and sat on the test instrument. They gradually reached forward as far as possible with their knees extended. The test was conducted twice, and the best of the two scores was retained (15).

#### Standing long jump

Standing long jump was conducted to assess lower-limb strength. Each subject stood at the starting line and was asked to jump forward as far as they could. The distance was measured in meters from the starting line to the heel of the closest foot. The test was conducted twice, and the best of the two scores was retained (15).

#### 800/1,000 m run

Each student stood at the starting line and was asked to complete the 800- or 1,000 m run as fast as they could. The time in minutes and seconds was recorded. Female students ran 800 m run, while male students ran 1,000 m (15).

#### Pull-ups

Pull-up was used to evaluate the upper body's muscular strength. The test was scored as the number of pull-ups. The subject jumped up and pulled the bars with both hands. After standing still, subjects pulled with both arms simultaneously. Only the male students performed this test (15).

#### Bent-leg sit-ups

Each subject was instructed to lay on a mat with knees bent at 90 degrees, raise their upper body, and touch their knees with their elbows. The number of bent-leg sit-ups completed in 1 min was recorded. Only the female students performed this test (15).

#### Physical fitness index

The specific calculation of the *Z*-score for each physical fitness test was (test value-national average)/national standard deviation; the shorter the time for the 50 m run, 1,000 m run for boys, and the 800 m run for girls, the better the performance. Therefore, the PFI was—Z pull-ups or 1-min sit-ups + *Z* standing long jump + *Z* seated forward bend-*Z* 50 m running-*Z* 1,000/800 m running (11, 22).

### Statistical analysis

Experimental data were processed using the IBM SPSS statistical software (version 26.0, Chicago, IL, USA). All data were presented as “mean ± standard deviation” (*M* ± SD). An independent sample *t*-test or one-way analysis of variance (ANOVA) was conducted to compare the mean differences among groups. When a significant interaction was observed, the LSD *post hoc* correction was performed to confirm the significance. The linear regression model in the Stata package was used to determine the trends in PFI throughout the study duration. The level of significance was set at *p* < 0.05 for all tests.

## Results

The one-way ANOVA models showed that PFI in the normal category was significantly higher as compared to all the other BMI categories in both male and female groups (*p* < 0.001), and PFI in the obese category was significantly lower as compared to all the other BMI category for males and females (*p* < 0.001). No significant effect on PFI for all the BMI categories was observed within the male and female groups (*p* > 0.150).

**Figure 1:**
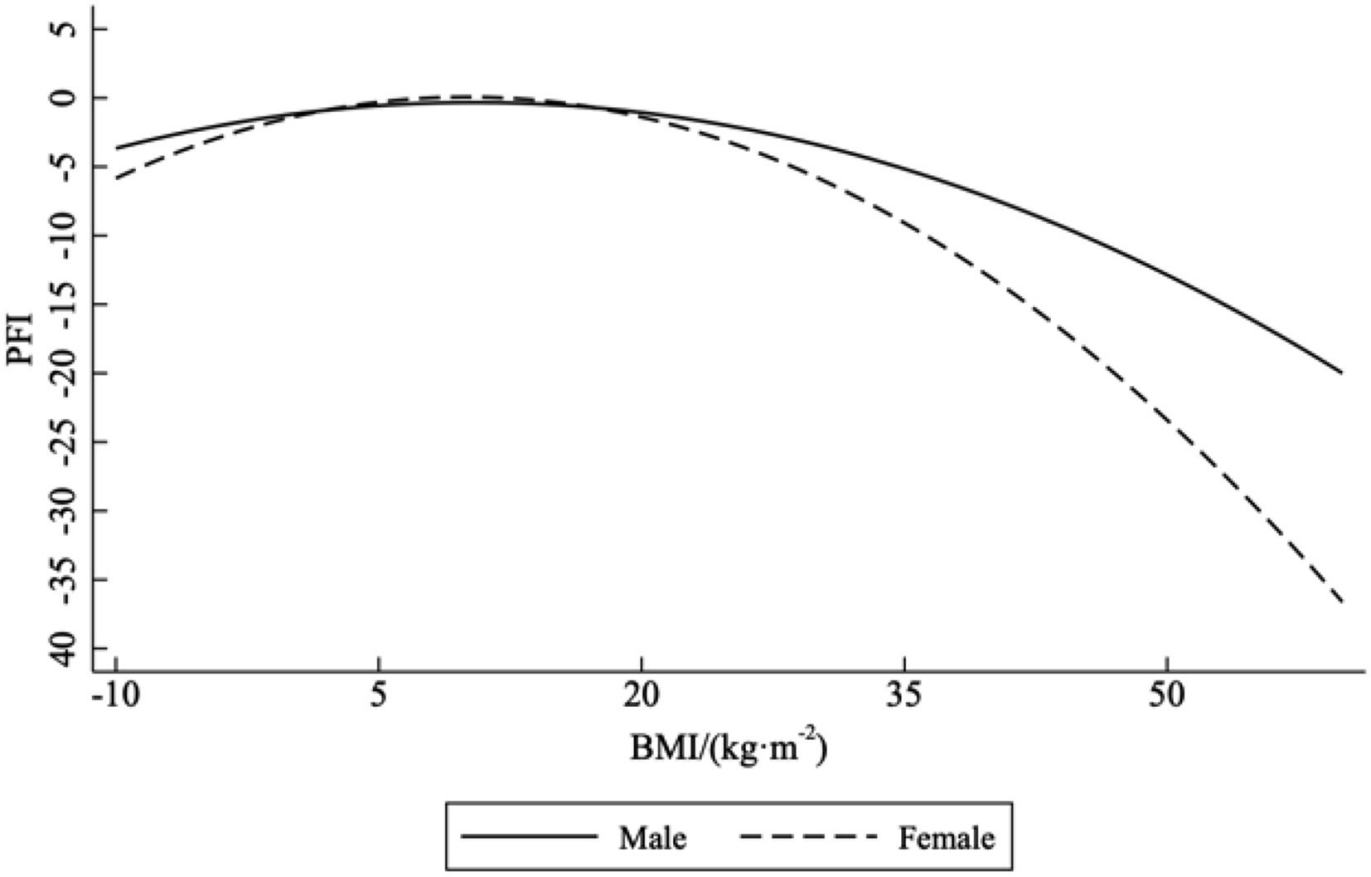
The relationship between PFI and BMI.

The logistic regression analysis showed that PFI in both males and females was related to BMI (*p* < 0.001). Figure 1 and Table 1 displays the relationship between PFI and BMI. The equations for gender-specific characteristics are as follows:


$$\begin{array}{l} PFI_{male}=-0.160BMI^{2}+0.135BMI-6.549\\ PFI_{female}=-0.294BMI^{2}+0.026BMI+1.405 \end{array}$$

**Table 1:** The percentage of BMI level grouped by gender and age.

**Gender**	**Age**	**Low**	**Normal**	**Overweight**	**Obesity**	***F***	**p**
Male high school students	16	0.64%	11.4%	3.25%	2.95%	37,789.3	< 0.001
	17	0.59%	10.99%	2.8%	2.43%	35,115.3	< 0.001
	18	0.65%	9.65%	1.99%	2.61%	33,032.9	< 0.001
Female high school students	16	0.55%	12.39%	2.26%	2.6%	320,707.9	< 0.001
	17	0.63%	12.49%	1.97%	1.93%	28,262.7	< 0.001
	18	0.69%	11.4%	1.45%	1.69%	24,097.4	< 0.001

**Figure 2:**
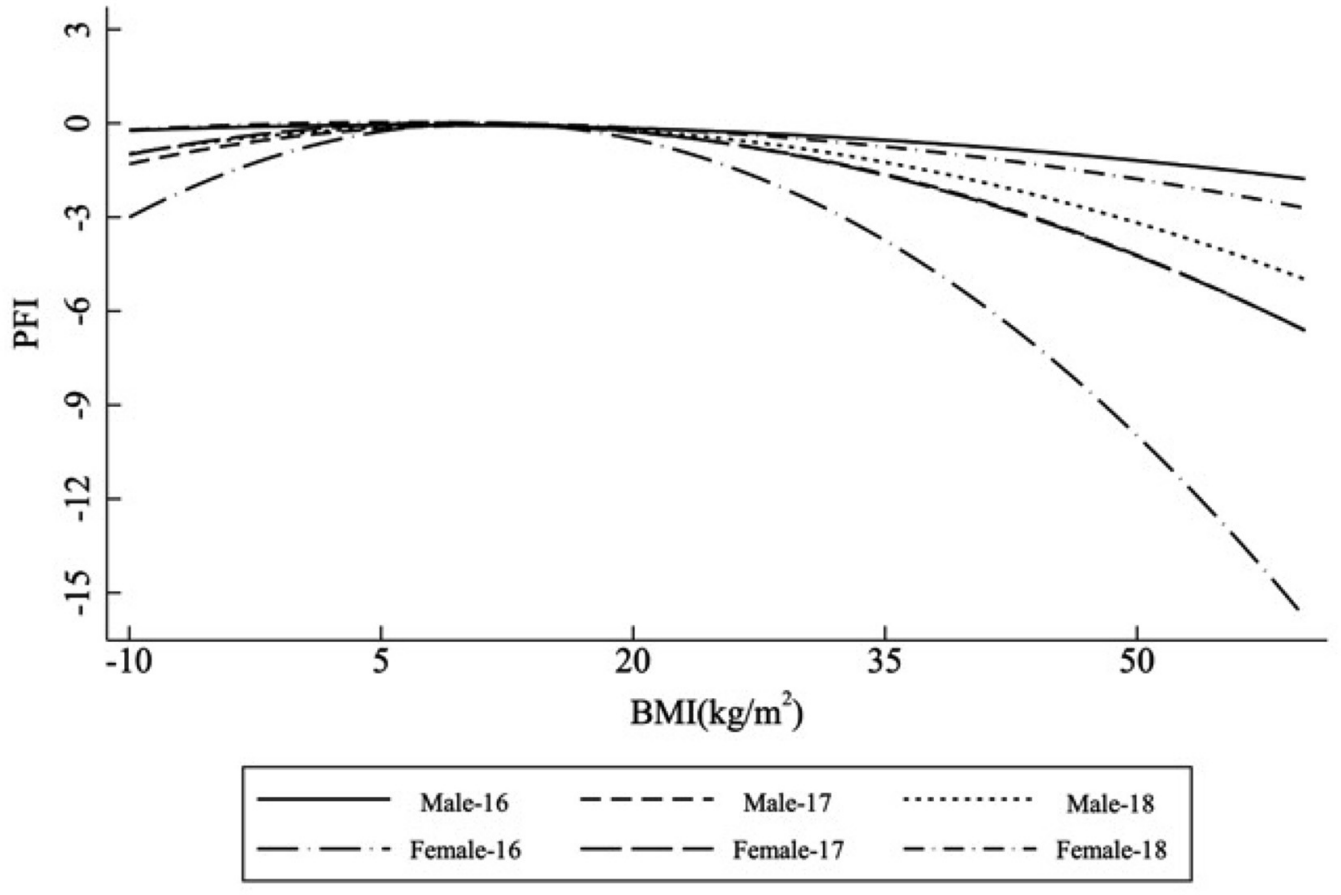
The relationship between PFI and BMI is grouped by gender and age.

Additionally, the logistic regression analysis showed that PFI in both males and females of different ages was related to BMI (*p* < 0.001). Figure 2 and Table 2 display the relationship between PFI and BMI in both males and females of different ages. The equations for age-specific characteristics are as follows:


$$\begin{array}{l} PFI_{male-16}=-0.016BMI^{2}-0.376BMI-6.249\\ \left(F=798.80,p<\;0.001\right).\\ PFI_{male-17}=-0.275BMI^{2}+0.642BMI-7.000\\ \left(F=885.10,p<\;0.001\right).\\ PFI_{male-18}=-0.205BMI^{2}-0.165BMI+1.722\\ \left(F=536.77,p<\;0.001\right).\\ PFI_{female-16}=-0.665BMI^{2}+1.673BMI-0.786\\ \left(F=959.38,p<\;0.001\right).\\ PFI_{female-17}=0.262BMI^{2}-0.165BMI+1.722\\ \left(F=793.40,p<\;0.001\right).\\ PFI_{female-18}=0.095BMI^{2}-0.708BMI+2.467\\ \left(F=464.08,p<\;0.001\right). \end{array}$$

**Table 2:** The PFI of participants grouped by gender, BMI, and age.

	**Low weight (numbers)**	**Normal (numbers)**	**Overweight (numbers)**	**Obesity (numbers)**	***F***	**p**
Male-16	−7.76 ± 1.40 (1,136)	−7.15 ± 1.45 (20,140)	−7.47 ± 1.48 (5,203)	−8.19 ± 1.53 (5,748)	782.50	< 0.001
Male-17	−7.33 ± 1.43 (1,036)	−6.76 ± 1.47 (19,414)	−7.15 ± 1.46 (4,284)	−7.76 ± 1.52 (4,945)	642.65	< 0.001
Male-18	−7.00 ± 1.53 (1,152)	−6.59 ± 1.39 (17,055)	−6.90 ± 1.40 (4,608)	−7.48 ± 1.51 (3,522)	412.40	< 0.001
Female-16	−0.85 ± 2.74 (972)	0.002 ± 2.35 (21,886)	−0.66 ± 2.30 (4,593)	−1.73 ± 2.38 (3,997)	661.26	< 0.001
Female-17	−0.37 ± 2.74 (1,121)	0.44 ± 2.25 (22,073)	−0.27 ± 2.15 (3,402)	−1.24 ± 2.19 (3,482)	621.60	< 0.001
Female-18	0.17 ± 2.73 (1,216)	0.76 ± 2.23 (20,136)	0.02 ± 2.10 (2,964)	−0.71 ± 2.22 (2,570)	393.41	< 0.001

## Discussion

This study demonstrated that BMI significantly affected PFI in both males and females. As compared to the obese and overweight categories according to the BMI, significantly higher scores of PFI were observed for males and females in the normal-weight group. Our results suggested that the relationship between PFI and BMI was non-linear, characterized by an inverted U-shape association. The results of this study suggested that the BMI of high school students in the normal category indicated greater physical fitness and good physical health; physical fitness became better and then worse with increased BMI.

Sports in schools are important to maintain physical fitness among high school students and the quality of school physical education must be improved. During childhood and adolescence, sports participation in childhood is linked to Health-Related Quality of Life (HRQoL) in young adulthood, whether it is in the form of individual or team sports, or an unstructured physical activity like backyard games (6). School physical education aims to encourage students to actively participate in physical exercise, develop the habit of exercising regularly, and improve their self-care ability and physical health (15). Physical health is also essential from a public health perspective (23). Physical fitness levels are strongly associated with health-related outcomes, including obesity, cardiovascular disease, bone health, mental health, and social psychology, which have good potential for physical fitness (24). Students in high school are under great learning pressure, leading to a significantly rising trend of obesity and an increased number of overweight individuals. Although genetic factors play an important role in obesity, environmental and lifestyle factors such as physical activity and nutrition patterns are also crucial (25). Previous studies show that this increasing trend may be attributed to rapid changes in dietary and physical activity patterns (26).

The results of this study suggested that both low weight and obese categories according to BMI would induce a negative effect on physical fitness and physical health levels, consistent with the findings of previous studies (10, 27–29). Ding and Jiang (30) found that overweight and obese students showed poorer performance in physical fitness tests as compared to their normal-weight counterparts irrespective of their sex. They also showed that in overweight and obese students additional load and restriction of movement caused by excess body mass further impeded their performance; energy requirements increase to perform physical activities with heavy loads as compared to those of normal-weight individuals, which can cause these students to avoid physical activity (30). According to our results, the relationship between BMI and PFI was characterized by an inverted U-shape association, similar to that described in a previous study. Normal weight students generally show better physical fitness than underweight, overweight, and obese students, especially among males (15).

Several limitations of the pilot study should be noted. First, this cross-sectional study cannot establish a causal relationship between physical fitness, body size, and fitness level, but it does identify an association between BMI and PFI. Second, the sample does not truly represent the number of high school students in China, as more than 95% of the study participants were from Jinan, Shandong Province. Additionally, though many of the observed results in physical fitness can be observed in these outcomes, body composition, and daily nutritional intake are more exact factors related to the level of physical fitness. Future studies consisting of body composition, food habits, and eating behaviors are thus demanded to confirm the findings of our study. Studies with larger sample sizes and comprising participants from different provinces, as well as other cohorts (e.g., age), are warranted to examine and confirm the observations in this study in the future.

## Conclusion

In conclusion, BMI affects the PFI in both males and females. Compared to the obese and overweight categories based on BMI, significantly higher scores of PFI were observed for males and females. Nevertheless, this study provided preliminary evidence that BMI affects the PFI in both males and females. Compared to the obese and overweight categories based on BMI, significantly higher scores of PFI were observed for males and females. Thus, PFI should be highly demanded to predict the physical fitness of high school students. Future prospective and longitudinal cohort studies must accurately identify the causal relations and potential mechanisms.

## Data availability statement

The original contributions presented in the study are included in the article/Supplementary material, further inquiries can be directed to the corresponding author.

## Ethics statement

The studies involving human participants were reviewed and approved by the Ethics Committee of Shandong Normal University (2021036). Written informed consent to participate in this study was provided by the participants' legal guardian/next of kin.

## Author contributions

GQ, BL, and YQ: design and/or conceptualization of the study. GQ and YQ: analysis and/or interpretation of the data. GQ drafting and/or revising the manuscript. All authors contributed to the article and approved the submitted version.
