# Supplementary figures and images for: Association between BMI and health-related physical fitness: A cross-sectional study in Chinese high school students
Source: Front Public Health. 2022 Dec 8;10:1047501. doi: 10.3389/fpubh.2022.1047501 (PMC9773132; doi:10.3389/fpubh.2022.1047501)

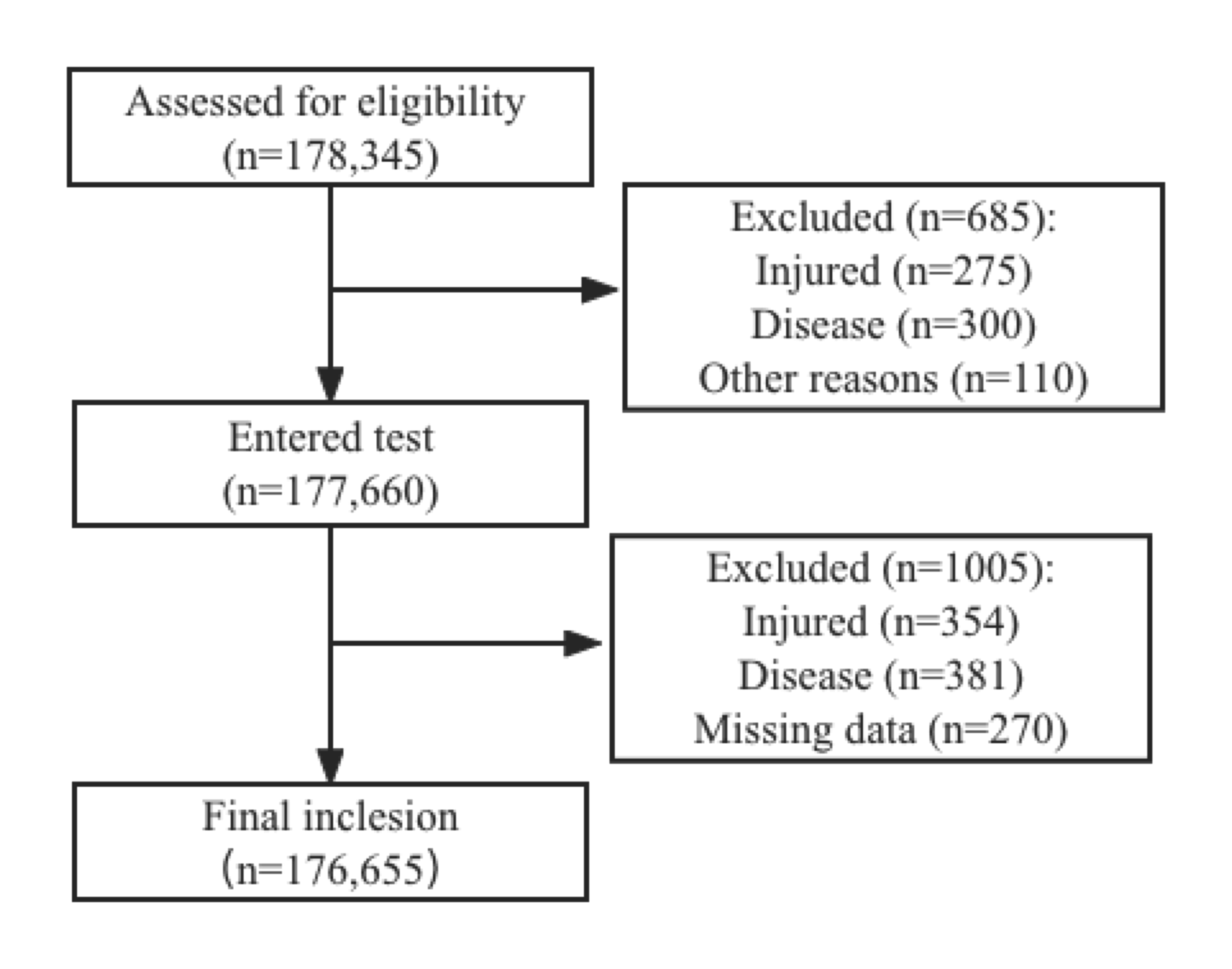


**Supplementary Figure 1.** The recruitment process.

Supplement: Supplementary file 1 [file Table_1.DOCX]
